# Ecological implications of reduced pollen deposition in alpine plants: a case study using a dominant cushion plant species

**DOI:** 10.12688/f1000research.4382.1

**Published:** 2014-06-19

**Authors:** Anya Reid, Robyn Hooper, Olivia Molenda, Christopher J. Lortie

**Affiliations:** 1 Department of Forest and Conservation Sciences, University of British Columbia, Vancouver, BC, V6T 1Z4, Canada; 2 Department of Chemical Engineering and Applied Chemistry, University of Toronto, Toronto, M5S 3E5, Canada; 3 Department of Biology, York University, Toronto, M3J 1P3, Canada

## Abstract

The reproductive assurance hypothesis states that self-incompatible female plants must produce twice the number of seeds relative to their self-compatible hermaphroditic counterparts to persist in gynodioecious populations. This is a viable life-history strategy, provided that pollination rates are sufficiently high. However, reduced pollination rates in alpine plants are likely due to climate induced plant-pollinator mismatches and general declines in pollinators. Using a gynodioecious population of the dominant plant *Silene acaulis* (Caryophyllaceae), we tested the reproductive assurance hypothesis and also the stress gradient hypothesis with a series of pollinator exclusion trials and extensive measurements of subsequent reproductive output (gender ratio, plant size, percent fruit-set, fruit weight, seeds per fruit, total seeds, seed weight, and seed germination). The reproductive assurance hypothesis was supported with female plants being more sensitive to and less likely to be viable under reductions in pollination rates. These findings are the first to show that the stress gradient hypothesis is also supported under a gradient of pollen supply instead of environmental limitations. Beneficiary abundance was negatively correlated to percent fruit-set under current pollen supply, but became positive under reduced pollen supply suggesting that there are important plant-plant-pollinator interactions related to reproduction in these alpine plant species.

## Introduction

At least two future climate change scenarios predict that pollination rates will be directly reduced, and these are due to either pollinator declines ( Potts *et al.*, 2010) and/or plant-pollinator mismatches ( Hegland *et al.*, 2009). Recently there has been concern over general global trends of reduced pollinator species abundance and diversity that are both predicted to reduce pollination rates to plants ( Memmott *et al.*, 2007; Potts *et al.*, 2010). Climate induced plant-pollinator mismatch can reduce pollination rates by creating a temporal mismatch in pollinator emergence and plant flowering times ( Hegland *et al.*, 2009). Pollinator emergence is regulated by temperature, whereas plant bloom time is regulated by photoperiod ( Hegland *et al.*, 2009). If climate warming shifts pollinator emergence but not plant bloom time, then a temporal mismatch between plants and pollinators occurs ( Hegland *et al.*, 2009). This scenario is likely more pronounced in alpine and polar environments that are experiencing a more rapid increase in annual temperature than the global average (very high confidence; Intergovernmental Panel on Climate Change [IPCC], 2013). Conceivably, both of these reductions in pollination rates occur simultaneously and thus adaptability of different sexual morphs in alpine plants can be an important consideration in predicting responsiveness and variation in reproductive output.

Hypotheses associated with pollen availability in alpine environments are controversial. It has been assumed that pollination rates are inherently low in alpine environments ( Larson & Barrett, 2000; Totland & Sottocornola, 2001; Torres-Díaz *et al.*, 2011). This is attributed to low temperatures, overcast conditions, strong winds, and relatively unpredictable weather being challenging for insect pollinators ( Körner, 1999). These harsh conditions generally lead to lower pollinator diversity, abundance, and activity in alpine ecosystems relative to milder ecosystems ( Kevan, 1972; Primack, 1978; Moldenke & Lincoln, 1979; Arroyo *et al.*, 1982; Primack, 1983; Billings, 1987; Totland, 1993). Alternatively, pollination rates can increase with elevations, suggesting adequate pollen availability under current conditions ( Arroyo *et al.*, 1985; Arroyo & Squeo, 1990; Bingham & Orthner, 1998; Utelli & Roy, 2000).

The reproductive assurance hypothesis (RAH) and the stress gradient hypothesis (SGH) are thus highly relevant hypotheses to explore in better understanding climate change effects on alpine communities. The RAH proposes that when pollen supply is low, self-compatible plants are favored over self-incompatible plants ( Lloyd, 1992; Lloyd & Schoen, 1992). This is because self-compatible plants create their own pollen thereby being more adapted to low or variable pollination rates ( Larson & Barrett, 2000; Muñoz & Arroyo, 2006; García-Camacho & Totland, 2009). Further, it has been proposed that self-compatible plants are less likely to become extinct if pollinators drastically decrease or disappear from a given system ( Richards, 1997; Morgan *et al.*, 2005). Therefore, self-compatible plants may become favored in the future if pollination supply declines. The SGH is also an important ecological theory to consider with respect to potential climate impacts on pollinators. The SGH states that facilitation between plant species is more common when resources are limited ( Bertness & Callaway, 1994). Typically, the SGH is tested using environmental limitations such as temperature or moisture in the alpine ( He *et al.*, 2013; Liczner & Lortie, 2014; McIntire & Fajardo, 2014), but has not been applied to the concept of pollen supply as an important limitation for plants in stressful environments. Taken together, these ecological theories provide a solid platform to build pollen limitation studies upon and also provide a set of potential ecological drivers that can help better predict pollination rate changes in the alpine.

Here, we use a gynodioecious population of *Silene acaulis* to assess the sensitivity of different genders to pollen limitation. We test the following predictions associated with the reproductive assurance hypothesis: (1) that the reproduction of self-incompatible plants is more sensitive to reduced pollen deposition than self-compatible plants and (2) that self-incompatible plants will be less viable under experimentally reduced pollen loads compared to self-compatible plants. In doing so, we also explore whether the SGH applies to the plant-pollinator system in the alpine. Specifically, we predict that facilitation between plants is more common under reduced pollen, i.e. that less pollen can be a novel stressor for alpine plants and that this can in turn relate to plant-plant interactions.

## Materials and methods

### Study species

*S. acaulis* (L.) Jacq. (Caryophyllacae), commonly known as moss campion, is a common long-lived evergreen cushion that is found throughout the northern hemisphere ( Hitchcock & Maguire, 1947). Each plant has a single strong taproot, and there is no clonal reproduction ( Morris & Doak, 1998). Small pink flowers can be abundant. *S. acaulis* is visited by bumblebees ( Shykoff, 1988; Shykoff, 1992; Marr, 1997; Delph *et al.*, 1999; Delph & Carroll, 2001), moths, beetles, ants ( Marr, 1997; Delph & Carroll, 2001), flies ( Totland, 1993; Delph & Carroll, 2001), butterflies and *Osmia* bees ( Reid & Lortie, 2012). *S. acaulis* is a nurse plant species that, like many other cushion forming plants, benefits other plant species (called beneficiaries) by reducing abiotic stress ( Arroyo *et al.*, 2003; Bertness & Callaway, 1994; Callaway & Walker, 1997; Cavieres *et al.*, 2006). Recent beneficiary removal studies suggest that by facilitating the beneficiaries, cushions bear a cost in reduced reproductive success ( Cranston *et al.*, 2012; Schöb *et al.*, 2014).

*S. acaulis* is sexually polymorphic ( Hitchcock & Maguire, 1947), and the population examined here was gynodioecious with plants that only have hermaphrodite flowers and other that only have female flowers. Female flowers have three styles with stigmatic lobes and hermaphrodite flowers have ten stamens. Male-sterility in female morphs of *S. acaulis* is predominantly under nuclear-cytoplasmic control ( Delph & Carroll, 2001); the gene for male-sterility is passed on through the female gamete ( Lewis, 1941). In addition to *S. acaulis,* the flowering plant species *Antennaria alpina*, *Arnica sp.*, *Carex* sp., *Erigeron sp.*, *Luzula* sp., *Phacelia sericea*, *Phlox diffusa*, *Phyllodoce* spp., *Poa alpina*, *Potentilla diversifolia*, *P. heptaphila*, *P. villosa*, *Ranunculus eschsoltzii*, *Saxifraga bronchialis*, and *Solidago multiradiata* were present at relatively high densities.

### Study site

The experiments were conducted on the Whistler Mountain in British Columbia 50°03′31.68″N, 122°57′22.53″W, 2168m elevation), Canada, during the snow-free season of July and August 2010. This area is classified as alpine tundra with ten months of snow cover per year ( Pojar *et al.*, 1987). A total of 273 *S. acaulis* plants were measured. Three *S. acaulis* plants were excluded from the study because they were infected with the pollinator-transmitted anther smut-fungus *Microbotryum violaceum* that renders the flowers of both genders sterile ( Baker, 1947; Alexander & Antonovics, 1988; Hermanutz & Innes, 1994; Marr, 1997).

### Treatments

Before bud-burst, *S. acaulis* plants were covered with cloth mesh to prevent pollinators contacting the flowers ( Donnelly *et al.*, 1998). As the plant gender was unknown when initially covering, 60 plants were covered to ensure that there would be sufficient replicate plants of each gender. Plant gender was established after bud-burst, and at that time, plants were randomly assigned a treatment such as covered with mesh or open to insect pollinators and marked with a unique identification code. Reduced pollination treatments were the ones covered with mesh and were applied to 20 hermaphrodite and 20 female plants. The first 20 female and male plants found were used as replicates with the additional 20 plants being uncovered. The 40 plants (20 of each gender) selected for the reduced pollination treatments remained covered with mesh for the entire flowering season to exclude all insect pollination.

All flowers of the reduced-pollination treatment plants were hand-pollinated with pollen collected from *S. acaulis* plants within 10 meters from the treatment plants. All hermaphrodite flowers with mature anthers were collected in the morning of the hand pollination days. Pollen was then applied using small paintbrushes or by directly touching the anthers to the stigmas of all the treatment-plant flowers. We found that direct contact of the anthers to the stigmas was the most effective method of hand pollination. The exact amount of pollen applied to each flower at each hand-pollination event was not quantified. Hand pollination was repeated on three different days between July 20 ^th^ and August 1 ^st^, 2010.

### Reproductive output measures

Reproductive output measures were collected from the 40 hand-pollinated treatment plants as well as 231 naturally pollinated *S. acaulis* plants. These measures included total number of flowers, total number of fruits, percent fruit-set, seeds per fruit, fruit weight, and seed weight. The percentage of germination was calculated on a subset of 60 plants, including the 40 treatment plants and 20 naturally pollinated plants.

The total number of flowers was counted during fruit collection including both successfully and unsuccessfully (i.e. no fruit) pollinated flowers. Fruits were collected when mature but not yet dehiscing, so that the seeds remained in the fruit capsule. This occurred between August 11 ^th^ and 25 ^th^. All fruits were placed in small labeled paper envelopes and were allowed to dry at room temperature to avoid decomposition. The percentage of fruit-set was calculated using the measures of total number of fruits and total number of flowers. The mean fruit weight (g) was calculated by averaging the weight of five randomly selected fruits per plant. These five fruits were dissected and the seed counted. The mean seed number per fruit was calculated from the seed counts. The total seed number per plant was estimated by multiplying total number of fruits with mean seed number per fruit. Mean seed weight (mg) was calculated by averaging the weight of ten randomly selected seeds per plant. All weighing was done to four significant digits. When a plant produced less than five fruits or ten seeds, the average was based on the maximum number of fruit or seed produced. Weighed seed was stored separately and cold-stratified at 4°C for two months. A test germination trial was conducted with limited success likely because the cold stratification was not sufficient. Therefore, seeds were then stored at 0°C for two additional months in preparation for germination trials.

Germination trials were conducted on the weighed and cold stratified seeds from the 40 *S. acaulis* plants in reduced pollination treatments and the remaining 20 labeled plants that were left open to natural pollination. Growth chambers were set to standard optimum growing conditions of 20°C and light for 12 hours, then 10°C and dark for the remaining 12 hours of the day ( Baskin & Baskin, 1998). Relative humidity was set to 90%. The ten seeds from an individual plant were placed on a labeled filter paper in a Petri dish. Seeds were checked weekly for three months, after which germination is rare ( Milbau *et al.*, 2009). Germination was considered to have occurred when the radical broke open the seed ( Milbau *et al.*, 2009). Germinated seeds were removed to speed-up counting during the subsequent weeks and reduce counting errors ( Milbau *et al.*, 2009). Percent germination was expressed as the fraction of total number of germinated seeds with respect to the total number of seeds per Petri dish.

Cushion area and floral density were measured because of their possible effect on reproductive output. Cushion area was defined by the external boundary of vegetation and calculated as an ellipse with the formula,

        cushion area = (a/2)*(b/2)*π

where *a* is the longest diameter of the plant and *b* the diameter perpendicular to *a*. We calculated the floral density by dividing the total flower number by the cushion area.

### Beneficiary abundance

To test if facilitation became more common under reduced pollen loads (stress gradient hypothesis), we measured beneficiary abundance on all cushion plants. Beneficiary abundance is the total number of individual plants living on the cushions.

### Statistical analysis

To assess the sensitivity of female and hermaphrodite reproductive output under reduced pollen deposition, we calculated the percent change of reproductive success measures within each gender between current and reduced pollen deposition. Percent change was calculated using the following equation:

        percent change = (T-C)/C*100

where T is the reproductive output measures under the reduced pollination treatments and C is the reproductive output measures under the current pollination rates ( Ayres, 1993). Negative numbers indicate that reduced pollination treatments decrease reproductive success and positive numbers indicate that reduced pollination treatments increase reproductive success. This method facilitates comparisons of the direction and magnitude of change.

To statistically test if reduced pollination, gender, and their interaction effects significantly explained the variation in the reproductive output measures, we used a generalized linear model (GLM) with Poisson distribution and a log link function. Covariate measures of *S. acaulis* included surface area, *S. acaulis* floral density and beneficiary abundance.

Hermaphrodites provide one-half of the genetic material to the population through pollen production. Therefore, female plants must produce at least twice the number of seeds as hermaphrodite plants to persist in the population ( Charnov, 1982) or have offspring that are more fit ( Lewis, 1941). To statistically analyze the viability of females under current and reduced pollen deposition levels, we compared female reproductive output to twice that of hermaphrodite reproductive output. In this way, if female reproduction (F) is greater than two times hermaphrodite reproduction (2H), then females are viable in the population. For females to be viable, not all measures of reproductive success need to be twice that of hermaphrodites, but all measures are shown to be comprehensive. GLMs were also used to test if gender significantly affected reproductive output measures under the current ambient and experimentally reduced pollination regimes.

Instead of testing the effect of beneficiaries on measures of reproductive success over a range of environmental gradients ( Bertness & Callaway, 1994) we tested whether variation in the current and experimentally reduced pollen supply can be viewed as potential stressor. To test the stress gradient hypothesis in this plant-pollinator system, we conducted correlation analysis and tested for significance in the interaction term between the effect pollination and beneficiary abundance on percent fruit set in a GLM. A significant p-value (p<0.05) indicates that the response of percent fruit set to beneficiary abundance significantly differs between plants in the current and reduce pollination regimes. All analyses are appropriate for dealing with the unbalanced number of replicates between the current and reduced pollination regimes and were done in JMP 10 ( SAS, 2012).

## Results

**Figure 1.**
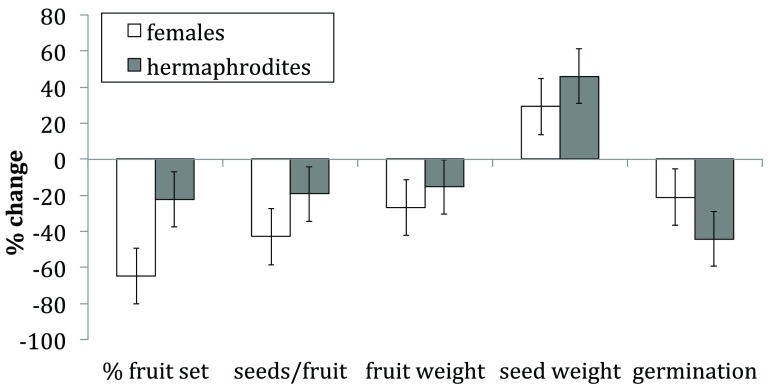
Percent change of female (white) and hermaphrodite (grey) reproductive output measures between current and reduced pollination regimes (p < 0.05). ± 1 standard error bars shown.

Pollination regime significantly influenced percent fruit-set, seeds per fruit, fruit weight and percent germination ( Table 1). Percent fruit-set, seeds per fruit, fruit weight, and percent germination decreased in both genders with reduced pollen deposition ( Figure 1). There was a significant interaction effect between gender and pollination regime for percent fruit set, and percent germination ( Table 1) indicating that these measures differed in their response. The direction of these differences was significant and is illustrated in the percent change calculations. Percent fruit-set was more significantly reduced in females relative to hermaphrodites ( Figure 1), whereas percent germination reduced to a greater in hermaphrodites compared to females ( Figure 1).

**Table 1.** Summary of GLM results testing the effect of gender, pollination regime, and the gender by pollination regime interaction on measures of reproductive success with covariate measures of *S. acaulis* surface area (SA), *S. acaulis* floral density, and beneficiary abundance indicated by *. Significance is considered at p < 0.05 and is indicated in bold.

		% fruit-set		seeds/fruit		fruit weight		seed weight		% germination	
Factor	DF	ChiSq	p>ChiSq	ChiSq	p>ChiSq	ChiSq	p>ChiSq	ChiSq	p>ChiSq	ChiSq	p>ChiSq
Gender	1	193.10	**<.0001**	6.92	**0.0085**	0.66	0.4163	0.81	0.3672	0.02	0.8865
Pollination regime	1	279.27	**<.0001**	32.43	**<.0001**	11.60	**0.0007**	1.32	0.2505	14.49	**0.0001**
Gender*pollination regime	1	240.93	**<.0001**	3.80	0.0513	0.31	0.5753	0.05	0.8213	40.02	**<.0001**
*S. acaulis* SA*	1	20.79	**<.0001**	6.89	**0.0087**	0.70	0.4022	0.21	0.6489	142.30	**<.0001**
*S. acaulis* floral density*	1	18.64	**<.0001**	0.16	0.6914	0.75	0.3862	0.20	0.6521	28.80	**<.0001**
Beneficiary abundance*	1	8.72	**0.0031**	1.12	0.2897	4.39	0.0362	3.96	**0.0466**	3.91	**0.0479**

**Figure 2.**
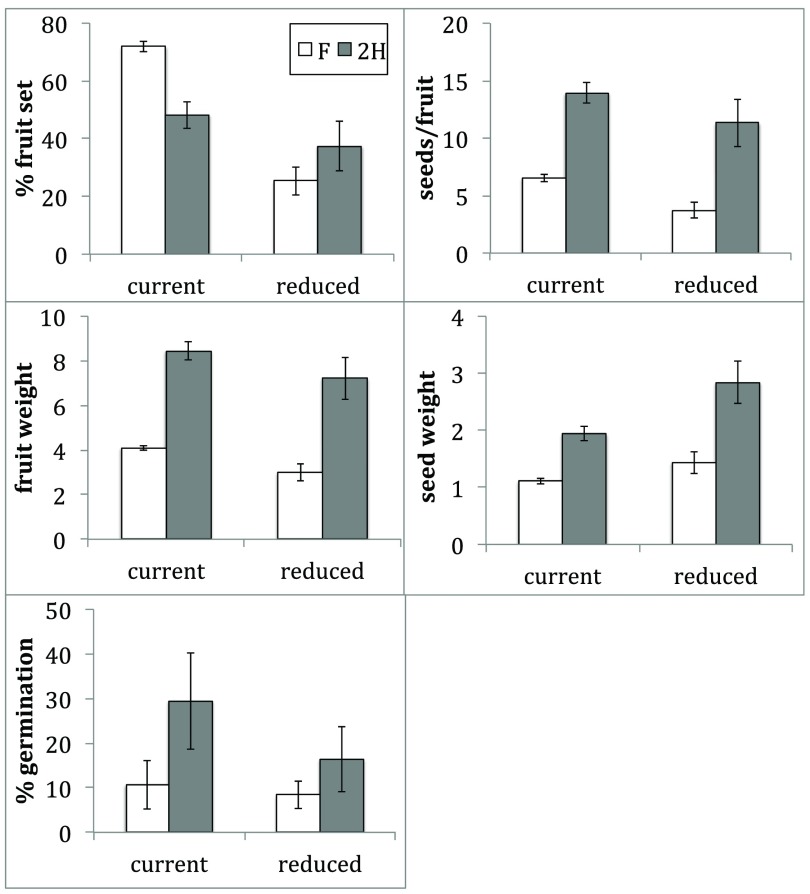
Reproductive output measures for female plants (white) and two times that of hermaphrodite plants (grey) under current and reduced pollination regimes (p < 0.05). ± 1 standard error bars shown.

Under current pollination rates, female plants had more than twice (2.98 times) the percent fruit-set compared to hermaphrodites ( Figure 2). Female plants had less than twice the seeds/fruit, fruit weight, seed weight and percent germination compared to hermaphrodites ( Figure 2). Under current pollination rates, all reproductive output measures were significantly different between females and two times hermaphrodite reproductive measures ( Table 2).

**Table 2.** Summary of GLM results testing the effect of gender on measures of reproductive success under the **current** pollination regime with covariate measures of *S. acaulis* surface area (SA), *S. acaulis* floral density, and beneficiary abundance indicated by *. Reproductive measures for hermaphrodites are doubled. Significance is considered at p < 0.05 and is indicated in bold.

		% fruit-set		seeds/fruit		fruit weight		seed weight		% germination	
Factor	DF	ChiSq	p>ChiSq	ChiSq	p>ChiSq	ChiSq	p>ChiSq	ChiSq	p>ChiSq	ChiSq	p>ChiSq
Gender (F and 2H)	1	394.83	**<.0001**	288.96	**<.0001**	159.83	**<.0001**	20.42	**<.0001**	68.09	**<.0001**
*S. acaulis* SA*	1	10.63	**0.0011**	6.64	**0.0099**	0.52	0.469	0.11	0.7395	13.05	**0.0003**
*S. acaulis* floral density*	1	28.74	**<.0001**	0.47	0.4912	0.31	0.5793	0.02	0.892	2.42	0.1198
Beneficiary abundance*	1	69.30	**<.0001**	1.82	0.1775	2.28	0.1313	6.89	**0.0087**	6.99	**0.0082**

Under reduced pollination rates, none of the female reproductive measures were greater than two times that of hermaphrodites ( Figure 2). Under reduced pollination rates, all reproductive output measures, except seed weight and percent germination, were significantly different between females and two times hermaphrodite reproductive measures ( Table 3).

**Table 3.** Summary of GLM results testing the effect of gender on measures of reproductive success under the **reduced** pollination regime with covariate measures of *S. acaulis* surface area (SA), *S. acaulis* floral density, and beneficiary abundance indicated by *. Reproductive measures for hermaphrodites are doubled. Significance is considered at p > 0.05 and indicated in bold.

		% fruit-set		seeds/fruit		fruit weight		seed weight		% germination	
Factor	DF	ChiSq	p>ChiSq	ChiSq	p>ChiSq	ChiSq	p>ChiSq	ChiSq	p>ChiSq	ChiSq	p>ChiSq
Gender (F and 2H)	1	54.79	**<.0001**	41.64	**<.0001**	15.03	**0.0001**	2.29	0.1298	3.45	0.0632
*S. acaulis* SA*	1	6.66	**0.0099**	3.30	0.0694	1.68	0.1945	2.78	0.0957	298.72	**<.0001**
*S. acaulis* floral density*	1	68.02	**<.0001**	24.68	**<.0001**	18.14	**<.0001**	3.74	0.053	71.77	**<.0001**
Ben. abundance*	1	61.84	**<.0001**	22.57	**<.0001**	8.62	**0.0033**	0.43	0.511	2.97	0.0847

**Figure 3.**
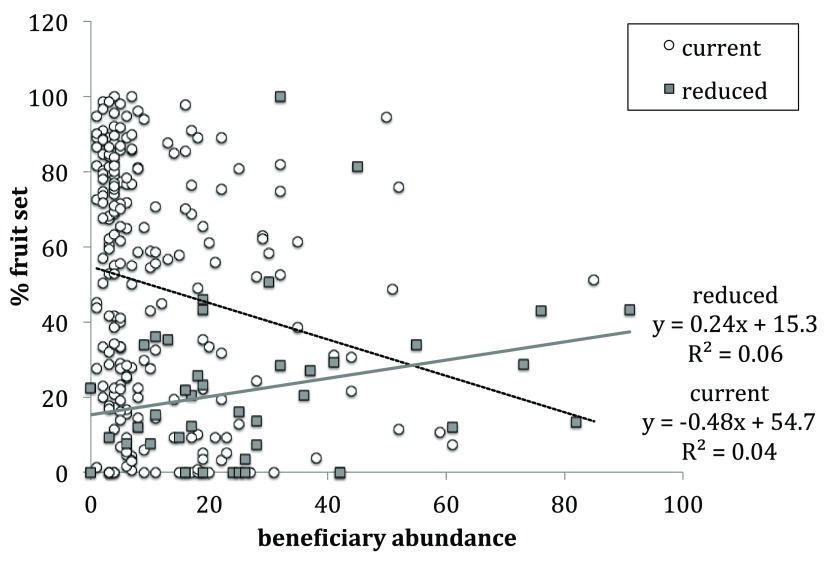
Percent fruit set and beneficiary abundance on individual *S. acaulis* plants under current pollination regime (white circles) and reduced pollination regime (grey squares). The linear best-fit line for plants under current pollination regime (black dashed line) has a negative slope of -0.48 and R ^2^ value of 0.04. The linear best-fit line for plants under reduced pollination regime (grey line) has a positive slope of 0.24 and R ^2^ value of 0.06. The slopes of these lines significantly differ (Chi ^2^ 161.25, p-value <0.0001).

Beneficiary abundance had a significant effect on percent fruit-set ( Table 1). Under the current pollination rates, percent fruit-set and beneficiary abundance are negatively related (slope = -0.48, R ^2^ = 0.04, Figure 3). In contrast, under reduced pollination rates, percent fruit-set and beneficiary abundance are positively related (slope = 0.24, R ^2^ = 0.06, Figure 3). The slopes of these lines significantly differ (Chi ^2^ 161.25, p-value <0.0001).

The reproductive effects of reduced pollen deposition via exclosures and hand pollination on the cushion plant *S. acaulis* *S. acaulis* is an important alpine plant species commonly demonstrated to have positive impacts on other plants and insects. In this experiment, we covered sets of plants (gynodioecious species with hermaphrodite and female flowers) with mesh with hand pollination only to examine the relative importance of pollen load as a potential stressor in alpine ecosystems.

## Discussion

Pollinator declines and climate mismatches are important in understanding the capacity for alpine plants to respond to possible future scenarios with reduced pollen loads. Using the cushion plant *S. acaulis*, we tested two predictions associated with the reproductive assurance hypothesis and more broadly we investigated whether pollination stress influences plant-plant interactions. All predictions were supported. Females were more sensitive than hermaphrodites to reduced pollen loads resulting in reproductive output dropping to below twice that of hermaphrodites. As the pollen supply conditions became more stressful (i.e. reduced), beneficiary plant species on these cushions positively related to the percent fruit-set of the cushions. Hence, the reproductive assurance hypothesis and use of pollen reduction experiments can be important tools for ecological experiments on the responsiveness of alpine plant-pollinator systems to future changes in pollen availability. Importantly, loss of keystone alpine plant species such as cushions may in turn have significant and reciprocal negative impacts on the pollinator communities.

Pollinator declines and plant-pollinator mismatches are important potential drivers of broad plant-community dynamics in the alpine if dominant cushion plant species are impacted because they often function as keystone plant species ( Arroyo *et al.*, 2003; Bertness & Callaway, 1994; Callaway & Walker, 1997; Cavieres *et al.*, 2006; Molenda *et al.*, 2012; Butterfield *et al.*, 2013; McIntire & Fajardo, 2014). Current trends of decreasing native pollinator populations are a pressing concern globally ( Memmott *et al.*, 2007; Potts *et al.*, 2010; Bartomeus & Winfree, 2013). In these alpine environments, bumblebees in particular are suggested to be critical because they are the most effective alpine pollinator in these ecosystems ( Bingham & Orthner, 1998; Chittka *et al.*, 1999; Gegear & Laverty, 1998), and for instance, because some alpine bumblebee populations are in decline ( Colla & Ratti, 2010). The future scenario that pollinators may emerge before flowers are in bloom due to a warming climate has also been proposed ( Hegland *et al.*, 2009) and shown in the alpine environments of Japan ( Kudo, 2013). Although it may not mimic the exact future pollen deposition rates, the experimental design tested herein begins to explore how alpine plants may respond to reduced-pollen loads. Differences in reproduction between alpine plant genders are thus a critical avenue of research and are important because cushion plants are common facilitators (for instance, see meta-analysis in Liczner & Lortie, 2014).

Two findings were particularly useful from an ecological perspective. Female cushion plants became less viable under reduced pollen loads and the stress gradient hypothesis was supported as means to model a gradient of pollen deposition rates because it is also clearly a potential limitation in some stressful ecosystems. Percent fruit-set was the only measure that indicated a drop in female viability. Indeed, this more than compensated for female plants’ reproductive disadvantage over hermaphrodite plants. Hence, a reasonable proxy or single measure to consider in similar future studies using dominant cushion plants is percent fruit-set only. Interestingly, our results also supported the application of the stress gradient hypothesis to pollen limitation in addition to its original formulation for environmental stress or consumer pressure. The relationship between beneficiary abundance and percent fruit-set shifted from negative to positive as the pollination rates were reduced. This supports previous findings that under current pollination to the plant community, beneficiary plants living on cushions generally have a cost associated with the cushion plants’ reproductive fitness ( Cranston *et al.*, 2012; Schöb *et al.*, 2013). The findings here however also further suggest that under reduced pollen loads this cost of facilitation can be diminished likely because competition between cushions and the other species is significantly reduced. Clearly, additional research is needed to identify the causal relationships between plant-plant interactions and plant-pollinator interactions with dominant plant species that host other species in stressful environments such as the alpine.

## Data availability

*F1000Research:* Dataset 1. The reproductive effects of reduced pollen deposition via exclosures and hand pollination on the cushion plant *Silene acaulis*, 10.5256/f1000research.4382.d29313 ( Reid *et al.*, 2014).
