# Supplementary material for: Ecological implications of reduced pollen deposition in alpine plants: a case study using a dominant cushion plant species
Source: F1000Res. 2014 Jun 19;3:130. [Version 1] doi: 10.12688/f1000research.4382.1 (PMC4103497; doi:10.12688/f1000research.4382.1)
Supplement: Peer review history

# Peer review: Referee response for version 1

**DOI:** 10.5256/f1000research.4691.r5194

**Published:** 2014-07-04

**Authors:** Ernesto Badano

**Affiliations:** 1 Division of Environmental Sciences, Potosinian Institute of Scientific and Technological Research (IPICYT), San Luis Potosi, Mexico

This is an interesting study dealing with the effects that facilitated plants may have on the performance of the reproductive success of the nurse species. As far as I am aware, this is the first study reporting such effects. The article is well written and is easy to read. The arguments provided in the introduction section support the hypotheses raised by the authors, and the methods used to test these hypotheses are sound. I believe that this paper makes an important contribution to the conceptual framework of positive interactions among plants along environmental gradients, mainly because it helps one to understand the outcome of these interactions in future climate change scenarios. I just have a few comments and queries about the methods used by the authors. The other sections of the article are clear and would not require major improvement.

**Materials and Methods**: Specific comments

1. In the subsection “Study species”, several sentences begin with the abbreviated scientific name of species ( *S. acaulis*). Nevertheless, it is not appropriate to use the abbreviated name of the species for beginning a sentence. In this case, the full name of the species should be provided ( *Silene acaulis*) while its abbreviated form ( *S. acaulis*) must be used when the species is mentioned in the middle of a sentence.
2. In the second paragraph of the subsection “Study species” the authors indicate that, besides *S. acaulis*, several other plant species are relatively abundant. My question on this issue is: Are all these other species beneficiary of *S. acaulis* or they are just sharing the habitat with this cushion plant? This should be clarified to understand the aim of testing the Stress Gradient Hypothesis.
3. In the first paragraph of the subsection “Treatments” the authors indicate that they choose 20 female and male plants for the experiment. This caught my attention because in the previous sections of the article the authors stated that they used a gynodioecious population of *S. acaulis* and, therefore, male plants should not exist. I think that there is a mistake in this sentence, where instead of “ *20 female and male plants*” the authors should say “20 female plants and plants with hermaphroditic flowers”.

I have read this submission. I believe that I have an appropriate level of expertise to confirm that it is of an acceptable scientific standard.

# Peer review: Referee response for version 1

**DOI:** 10.5256/f1000research.4691.r5192

**Published:** 2014-07-02

**Authors:** He Qiang

**Affiliations:** 1 School of Environment, State Key Laboratory of Water Environment Simulation, Beijing Normal University, Beijing, China

This is a well conducted experiment and well written manuscript. It is the first experimental test of the stress-gradient hypothesis under pollen limitation stress, showing how we can further our understanding of plant-plant interactions with a new perspective. In my view, this is a neat paper, though I have some concerns that I hope the author can address in a revision.

The main limitation of this paper is that plant-plant interactions are inferred by association, not experimental removal of neighbours. Although a number of studies from alpine habitats suggest that facilitation can be indicated by positive association relationships, this limitation I feel should be clarified in the paper.

It is also a bit confusing to me why the authors did hand pollination for reduced-pollination treatment plants - was this because reduced-pollination treatment plants did not receive pollination at all without hand pollination? If so, the question would be how the authors controlled the magnitude of hand pollination as compared to natural pollination.

A third issue is that the pollen reduction treatments lacked procedural control. For example, one cannot tell the effect of mesh on the data. Also surely, the authors need to provide some details about the cloth mesh they used, such as mesh size etc.

It is unclear what can be the potential mechanisms for facilitation under pollen limitation. Facilitation in physically stressful habitats is produced by neighbours’ relief of abiotic stress, and in biologically stressful habitats by associational defence. It is not a must for the authors to experimentally demonstrate this in the paper but I think they should at least discuss some of the possibilities. The authors may also want to include descriptions to encourage future research that experimentally tests for these possibilities.

I have several more specific comments:

1. " *Before bud-burst ...*" Clarify the start and end dates of your experimental treatments. What is the total duration?
2. " *The first 20 female and male plants found were ...*" Does the male here mean "hermaphrodite"? Use terms consistently.
3. For treatments, how many plants in current pollination treatments are female and male, respectively?
4. " *Reproductive output measures were collected from the 40 ...*" 40 hand pollinated, 231 naturally pollinated, this is 271 in total. So this number is inconsistent with the numbers described above: 273 plants were measured. There are three plants that were excluded?
5. Clarify when the beneficiary abundance was quantified and how this was done. It is the total number of plants living within the cushion. Shouldn't it be density per unit area?
6. How many beneficiary species occurred within your 273 study cushions? Would it be possible that beneficiary diversity rather than abundance is more likely to affect cushion plant reproductive success?
7. Actually, the beneficiary increased cushion plant fruit set under reduced pollen limitation. This means the beneficiary was actually a benefactor. This is a bit confusing; might it use neighbours instead?
8. " *we used a generalized linear model (GLM) with Poisson distribution ...*" Poisson distribution is often used for positive integer response variables. How about over-dispersion? Over-dispersion can substantially affect significance tests.
9. " *Under the current pollination rates, percent fruit-set and beneficiary abundance are negatively related ...*" Did pollination reduction affect the relationship between other reproductive variables and beneficiary abundance?

Overall, this is a great paper. The publication of this paper can be a stimulator of a new direction of positive species interactions with environmental stress.

I have read this submission. I believe that I have an appropriate level of expertise to confirm that it is of an acceptable scientific standard.

# Peer review: Referee response for version 1

**DOI:** 10.5256/f1000research.4691.r5191

**Published:** 2014-07-02

**Authors:** Fabio Bulleri

**Affiliations:** 1 Dipartimento di Biologia, Università di Pisa, Pisa, Italy

This is an interesting article reporting on a study investigating how reduced pollen supply influences reproduction of self-compatible versus self-incompatible plants of an alpine cushion species and its interaction with associated species. The main strength of the study is that of providing hints on the potential role that factors other than environmental conditions can play in modulating species interactions. In a relatively recent work by Maestre *et al.* (2009), stress has been divided into two major types, resource and non-resource. I am not sure whether pollen availability can be seen as a resource, but I would not have major issues with viewing pollinator availability as a quantifiable, limiting resource. Maybe, these concepts could be touched upon somewhere in the Introduction.

The main weakness of the study is the lack of quantification of pollen delivery in different treatments: I think the authors should try to give the reader, at the very least, a gross idea of the order of magnitude of differences between natural and artificial pollen supply. The second point is that there is not a formal gradient of pollen supply, as this implies more than two levels. This does not impinge on the robustness of the results, but limits the insight the study offers into the Stress Gradient Hypothesis.

Finally, both in the Abstract and M&M I did not get clearly who the benefactors and beneficiary were. Actually, only from the Results did I understand that you were analyzing the effects of species hosted by the cushion on the cushion-forming species. I sense that referring to the cushion and associated species might be a better solution.

**SPECIFIC COMMENTS**

**Introduction**

- It is not clear what grounds the alternative model for pollinator abundance (increasing with elevation) in alpine environments builds upon.
- In the Introduction, you refer to the stress source sometimes as ‘pollination supply’ and sometimes as ‘reduced pollen’ or ‘less pollen’. It would be useful to specify if the limiting resource is pollen supply (which implies reduced production by plants) or pollinator abundance (I guess the second, from the model you propose in the Introduction). 
- Is there any evidence of competition between hermaphrodite and female plants when pollen is not limiting?

 

**Material & Methods**

- You need to be consistent in the way you refer to hermaphrodite plants.
- Some plants could receive pollen from insects while others were covered with mesh and hand-pollinated. It is not clear how insect versus hand pollination generated a gradient of pollen supply. Do you assume that delivery of pollen by insect was much higher? If so, which order of magnitude?
- “ *Instead of testing the effect of beneficiaries on measures of reproductive success over a range of environmental gradients (Bertness & Callaway, 1994) we tested whether variation in the current and experimentally reduced pollen supply can be viewed as potential stressor*.” Again, it is not clear here who is expected to be facilitated and by whom (see my general comments). Likely, it would be useful to be more specific in your predictions at the end of the Introduction. Also, switch beneficiary and % fruit set in the last sentence of the Abstract (the way it is, it suggests that you are mainly looking at effect of fruit set on beneficiary abundance).

**Results**

- Estimates of effects could be reported in Tables, so as to give an idea of their direction.
- The relationship with beneficiary abundance explains little variation in % fruit set (Fig.  3), suggesting that other factors are of overwhelming importance. This point should be made clear in the Discussion.

**Discussion**

- It would be interesting if the authors could offer a possible explanation for a positive relationship between beneficiary abundance and % fruit set under reduced pollen supply. What are the potential mechanisms?

I have read this submission. I believe that I have an appropriate level of expertise to confirm that it is of an acceptable scientific standard, however I have significant reservations, as outlined above.
